# First Report of *Acanthamoeba* Genotype T4 from the Newly Formed Tajogaite Volcano Tephra (La Palma, Canary Islands)

**DOI:** 10.3390/pathogens13080626

**Published:** 2024-07-27

**Authors:** Patricia Pérez-Pérez, María Reyes-Batlle, Rubén L. Rodríguez-Expósito, Adolfo Perdomo-González, Ines Sifaoui, Francisco J. Díaz-Peña, Rodrigo Morchón, Sutherland K. Maciver, José E. Piñero, Jacob Lorenzo-Morales

**Affiliations:** 1 Instituto Universitario de Enfermedades Tropicales y Salud Pública de Canarias (IUETSPC), Universidad de La Laguna (ULL), 38200 San Cristóbal de La Laguna, Spain; mresyeba@ull.edu.es (M.R.-B.); rrodrige@ull.edu.es (R.L.R.-E.); isifaoui@ull.edu.es (I.S.); jpinero@ull.edu.es (J.E.P.); jmlorenz@ull.edu.es (J.L.-M.); 2 Departamento de Obstetricia y Ginecología, Pediatría, Medicina Preventiva y Salud Pública, Toxicología, Medicina Legal y Forense y Parasitología, Universidad de La Laguna (ULL), 38200 San Cristóbal de La Laguna, Spain; 3 Centro de Investigación Biomédica en Red de Enfermedades Infecciosas (CIBERINFEC), Instituto de Salud Carlos III, 28029 Madrid, Spain; 4 Departamento de Biología Animal, Edafología y Geología, Universidad de La Laguna, 38200 San Cristóbal de La Laguna, Spain; apgonzalez@ull.edu.es (A.P.-G.); fjdiazpe@ull.edu.es (F.J.D.-P.); 5 Zoonotic Infections and One Health GIR, Laboratory of Parasitology, Faculty of Pharmacy, University of Salamanca, 37008 Salamanca, Spain; rmorgar@usal.es; 6 Centre for Discovery Brain Sciences, Edinburgh Medical School, Biomedical Sciences, University of Edinburgh, Hugh Robson Building, George Square, Edinburgh EH8 9XD, UK; sutherland.maciver@ed.ac.uk

**Keywords:** *Acanthamoeba*, tajogaite, La Palma, tephra, pathogenic potential

## Abstract

The Tajogaite Volcano erupted on the western slope of the Cumbre Vieja mountain range on La Palma Island in the Canary Islands, Spain, in 2021. As one of the multiple consequences of this eruption, a layer of tephra was deposited, to a variable extent, over a large part of the island. Tephra deposits affect all aspects of vegetation recovery, the water cycle, and the long-term availability of volcanic nutrients. Protozoa, including free-living amoeba (FLA), are known to be among the first microorganisms capable of colonizing harsh environments. In the present study, the presence of FLA has been evaluated in the Tajogaite Volcano deposits. Samples of the tephra were collected and incubated at 26 °C on 2% non-nutrient agar plates with a layer of heat-killed *E. coli*. Morphological features, as well as the DF3 region sequence of the 18S rDNA, confirmed the presence of a T4 genotype strain of *Acanthamoeba.* Thermotolerance and osmotolerance assays were used to evaluate the strain’s pathogenic potential. This strain was considered thermotolerant but poorly osmotolerant. To the best of our knowledge, this is the first report of *Acanthamoeba* being isolated from a recently erupted volcano.

## 1. Introduction

The Canary Islands archipelago is located on a passive continental margin which extends parallel to the NW African continental platform. This archipelago is made up of eight islands: La Palma, El Hierro, La Gomera, Tenerife, Gran Canaria, Lanzarote, Fuerteventura, and La Graciosa [1]. The age of the islands, which are volcanic in nature, ranges from roughly 21 million years (Myr) to 0.8 Myr [2]. The Canaries are also characterized by a broad variety of topological and climatological variability, which has resulted in vegetation zones that range from semi-desert succulent scrub (0–700 m) to pine forests (1200–2000 m) and montane scrub (1900–2500 m) [3]. Due to the influence of the NNE trade winds and the subtropical North Atlantic’s relatively cool waters, the Canary Islands experience a temperate climate [4].

**Figure 1:**
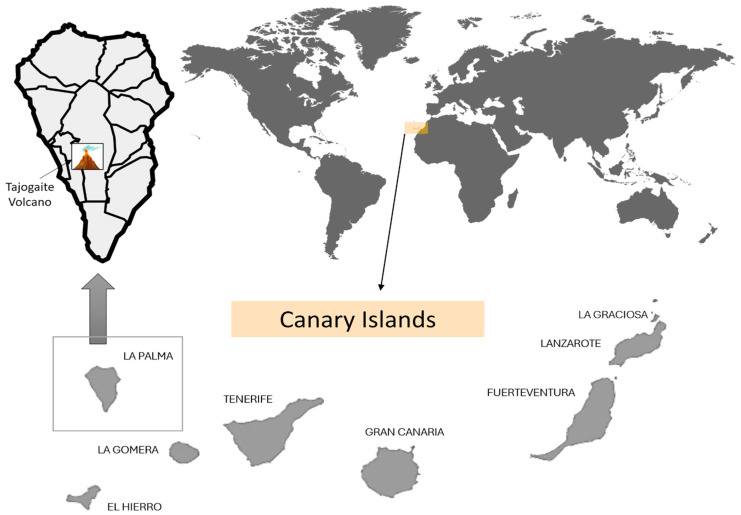
The island of La Palma and the geographical localization of the Tajogaite volcano. The sampling was collected from the El Paso municipality (28°37′38.2″ N, 17°52′24.1″ W) and geolocated via the Google Maps GPS tool.

La Palma Island, with 708 km2 of surface area and around 85,000 inhabitants, is the most north-westerly of the Canary Islands, and it is characterized by a mountain range which divides the island from north to south [5]. It has a warm climate that is sunny for most of the year, with rainfall occurring in the fall and winter, according to Miller et al. (2020) [6]. The height and dry northwest winds, along with the humid northeast trade winds, create an inversion layer that gives rise to a laurel forest with rich floral diversity [6] (Figure 1). On 19 September 2021, on the small island of La Palma, an explosion began an 85-day eruption, pouring 215 million m3 of lava onto the SW slope of the Cumbre Vieja Natural Reserve [5]. At the time of its eruption, the Tajogaite volcano had an altitude of about 1100 m, and it is currently 1120 m [7]. The eruption started with an early explosive phase that sent ash clouds to over 5000 m elevation, followed by rapidly advancing lava flows alongside violent strombolian activity [8]. This eruption, which affected densely populated areas of the island and lasted 85 days, caused more than 800 million euros of damage and necessitated the evacuation of more than 7000 people [9]. 

Environments related to volcanic activity are diverse, from deep ocean basaltic habitats to acidic hot springs, and are widely distributed on Earth. Therefore, it is of geomicrobiological importance to understand the diversity and characteristics of the microbial life that they harbour [10]. Free-living amoebae (FLA) are a group of unicellular organisms that have a great ability to grow in different environments in nature, such as water, soil, and dust. Although they can be found freely in the environment, they can cause infections in animals and humans [11]. For this reason, they are considered amphizoic [12]. Among the great variety of existing free-living amoebae, *Acanthamoeba* spp., *Naegleria fowleri*, *Balamuthia mandrillaris*, *Sappinia pedata*/*diploidea*, *Vahlkampfia* spp., and *Vermamoeba vermiformis* are known to infect humans [13,14,15]. These infections include an amoebic encephalitis known as primary amoebic meningoencephalitis (PAM), which is caused by *Naegleria fowleri*; granulomatous amoebic encephalitis (GAE), caused by the genus *Acanthamoeba* together with *Balamuthia mandrillaris*; and amoebic encephalitis, caused by *Sappinia pedata*. Other infections are keratitis caused mainly by the genera *Acanthamoeba* and *Vahlkampfia* and, less frequently, *Vermamoeba vermiformis* [13,14,16]. On the other hand, current molecular techniques, especially the sequencing of 18S rRNA genes, are being used to understand the species complex and the phylogeny of *Acanthamoeba*. Based on sequence differences, 23 genotypes (T1–T23) of *Acanthamoeba* have been established. Each genotype exhibits 5% or more sequence divergence between different genotypes [17,18,19,20]. In addition, FLA have been reported as vehicles for other microorganisms, including bacteria, fungi, and viruses [21,22]. These various characteristics of FLA mean that they are of great importance to human and environmental health [23].

These opportunistic parasites have been reported in the Macaronesian islands, including the Canary Islands. They are present in many of these volcanic islands, such as El Hierro, Gran Canaria, and Tenerife, among others. They have been found in waters of different sources and soils [24,25]. In contrast, FLA have never been described from recent volcanic ashes in these islands. Due to the exclusion zone set up around the volcano established by the authorities, we were not able to obtain lava samples. However, abundant tephra deposits were sampled in the municipality of El Paso. Tephra is considered to be a volcanic material, as scoria, dust, etc., are ejected during an eruption. In order to elucidate the capacity of FLA to colonize a newly formed volcanic soil, the aim of this study was to incubate a tephra sample from the Tajogaite volcano in a specific FLA agar medium. We also characterized the physicochemical nature of the tephra.

## 2. Materials and Methods

### 2.1. Location and Sampling

**Figure 2:**
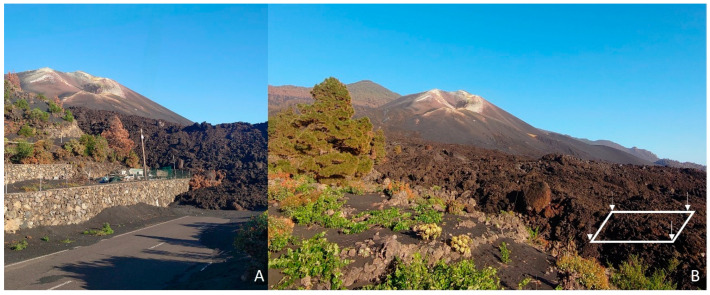
General view of the sampling site where the lava flow solidified after cooling is observed (**A**). The samples were collected (four white arrows) directly from the last accessible lava flow site (**B**).

Sampling was conducted on the volcanic island of La Palma (Canary Islands, Spain) (Figure 1). The tephra sample was collected in the municipality of El Paso (28°37′38.2″ N, 17°52′24.1″ W) during the month of June 2022, 6 months after the end of the eruption. In order to obtain homogenous samples from the sampling site, approximately 1 g from 4 different equidistant soil spots were taken. The sampling point was the closest site to the volcanic cone the public was able to access (Figure 2). Samples were kept at 4 °C until further processing in the laboratory. 

### 2.2. Tephra Sample Characterization

The particle size distribution was evaluated by sieving 100 g of the previously dried tephra for 5 min using an analytical sieve shaker operating at an amplitude of 2.0 mm through a standard series of 10 sieves with mesh sizes of 8, 4, 2, 1, 0.5, 0.25, 0.125, 0.063, and 0.032 mm. The textural group and sample statistics (median, mean, sorting) were calculated using GRADISTAT version 9.1 software. The pH and electrical conductivity (EC) were determined in a 1:5 aqueous extract (10 g of tephra in 50 mL of distilled water). Oxidizable organic carbon was measured via potassium dichromate oxidation and subsequent spectrophotometric measurement; total nitrogen content (N) was measured via dry combustion and the measurement of the resulting gases with a thermoconductivity cell from the LECO CN828 equipment. The total contents of Si, Ca, Mg, Na, K, S, P, Fe, Mn, Cu, Zn, Ni, Cr, Cd, Mo, Co, Pb, and B were determined after acid digestion of the sample via inductively coupled plasma (ICP).

### 2.3. Free-Living Amoeba Isolation

**Figure 3:**
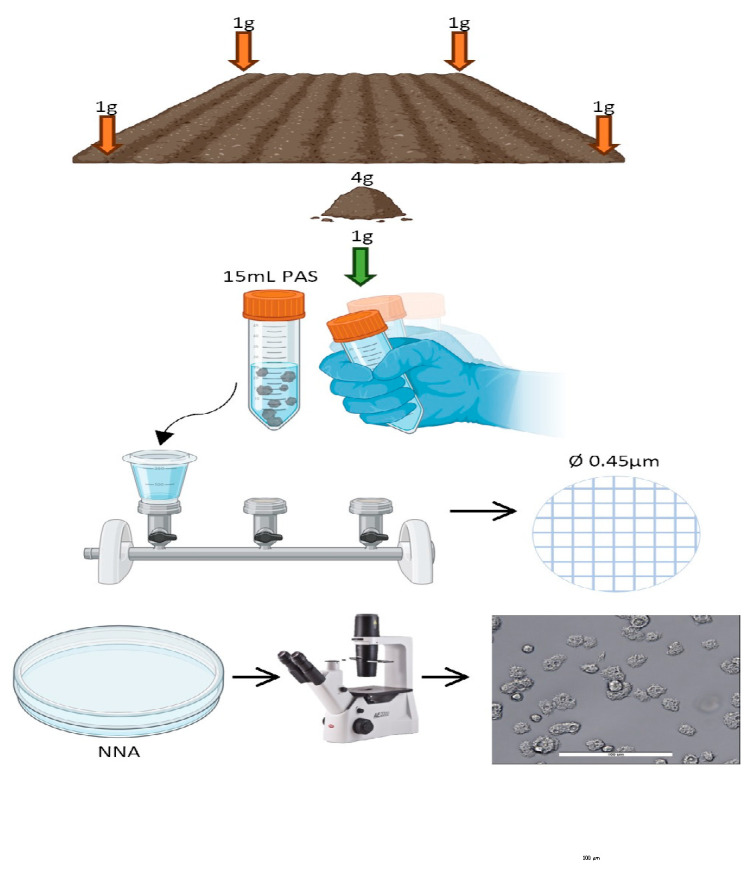
Workflow scheme for the sample processing. A total of 4 equal g of tephra were collected, homogenized, and mixed with 15 mL of PAS. The supernatant was filtered through a nitrocellulose membrane (Ø 0.45 µm) by a vacuum pump. The filter was then seeded and inverted onto an NNA plate and monitored daily for the FLA search.

The tephra sample was processed in this way: the 4 g of tephra were homogenized in a sterile glass, and then 1 g was resuspended in 15 mL of Page’s amoeba solution (PAS). After vortexing it, the supernatant was filtered by a vacuum filtration system using a 0.45 μm pore size filter (Pall, Madrid, Spain). The filter was then cultured inverted onto a non-nutrient agar (NNA) plate and incubated at 26 °C with a layer of heat-killed *E. coli*. The plate was visualized daily to check the presence of FLA. When the presence of trophozoites and cysts of FLA was observed, following the morphological characteristics of Page’s keys [26,27], we selected the growth spot and cloned it via dilution in a fresh NNA plate until a monoxenic culture was obtained (Figure 3). The isolated amoeba was then transferred and grown in PYG ATCC712 liquid culture medium and supplemented with 10 mg/mL of gentamicin and grown axenically thereafter.

### 2.4. Tolerance Assays

Assays for thermal and osmotic toleration were carried out as previously described [28], with some modifications. For the osmotolerance assays, a 10^3^ cells/mL suspension from the axenic culture was seeded as a 100 µL spot in the centre of an NNA plate and a layer of heat-killed *E. coli* containing mannitol 0.1 M, 1.0 M, and 1.5 M. Then, they were monitored daily for the presence of trophozoites and cysts for one week. With respect to thermotolerance assays, we repeat the process described above; but in this case, the plates were incubated at 26 °C, 37 °C, and 42 °C and monitored daily up to one week. To be able to compare and quantify our findings, we have established a “+” scale based on the growth of trophozoites/cysts on the plate.

### 2.5. DNA Extraction

For DNA extraction, the following steps were followed: 4 mL of Page’s amoebae solution (PAS) was added to the plate with the monoxenic amoebae culture. The plate was scraped, this suspension was centrifuged, and the concentrated amoeba culture was introduced directly into the Maxwell^®^ 16 tissue DNA purification kit sample cartridge (Promega, Madrid, Spain) following the manufacturer’s instructions, as has been previously described [29]. Amoebic genomic DNA yield and purity were determined using the DS-11 Spectro-photometer (DeNovix^®^, Wilmington, NC, USA).

### 2.6. PCR and Molecular Characterizations

PCR amplification of the 18S rRNA gene from the extracted DNA was carried out using specific primers for *Acanthamoeba* genus: JDP-1f 5′-GGCCCAGATCGTTTACCGTGAA-3′ and JDP-2r 5′-TCTCACAAGCTGCTAGGGAGTCA-3′ [30] (Tm = 50 °C). PCR reactions were performed in a 50 μL mixture containing 40 ng of DNA yield, and the PCRs were performed in 35 cycles with denaturation (95 °C, 30 s), annealing (60 °C, 30 s), and primer extension (72 °C, 30 s). After the last cycles, the primer extension was maintained for 7 min at 72 °C. The expected amplicon length varies at 500 bp for JDP. Amplification products from all PCRs were analyzed via electrophoresis through a 2% agarose gel, and positive PCR products were sequenced using Macrogen Spain service (Madrid, Spain).

### 2.7. Phylogenetic Analysis

In order to establish a genetic relationship with reference strains, a sequence alignment was performed using the ClustalW software 2.0. The MEGA 11 program and the maximum likelihood method were used to infer the evolutionary history [31,32]. The species identification has been based on sequence homology analysis compared with the available DNA sequences in the GenBank database.

## 3. Results

### 3.1. Tephra Samples Characterization

The values of the physicochemical variables analyzed in the tephra sample are given in Table 1.

The recently erupted material from the Tajogaite volcano on La Palma Island exhibits distinct physical properties, particularly in terms of its porosity and ability to retain water. The high percentages of sand (63.5%) and gravel (36.2%) suggest a generally coarse texture (textural group = sandy gravel; median or D50 = 1231 µm; geometric mean = 1186 µm; sorting = poorly sorted), which, in turn, indicates a relatively high porosity. This porosity, while beneficial for drainage, likely limits water retention due to the minimal presence of fine particles, as indicated by the very low percentage of silt (0.3%). The electrical conductivity of 0.1 dS m^−1^ and pH of 6.9 indicate moderate conditions, neither strongly acidic nor alkaline, yet the moisture retention capability might be compromised, challenging the colonization and survival of organisms, including the free-living amoebae.

### 3.2. FLA Presence Detection

**Figure 4:**
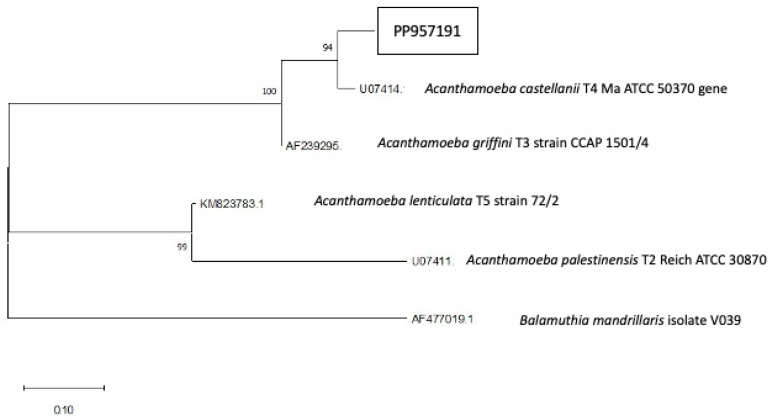
Initial tree(s) for the heuristic search were obtained automatically by applying Neighbor-Join and BioNJ algorithms to a matrix of pairwise distances estimated using the Tamura-Nei model, and then selecting the topology with superior log likelihood value. This analysis involved six nucleotide sequences. There were a total of 2388 positions in the final dataset. Evolutionary analyses were conducted in MEGA11 [31].

**Figure 5:**
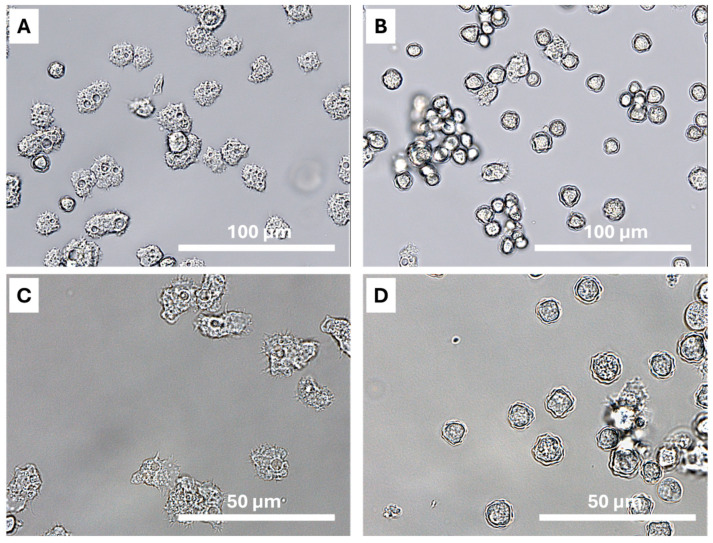
Micrographs of *Acanthamoeba* sp. T4 from the Tajogaite tephra sample in PYG ATCC712 liquid culture medium: Trophozoites (**A**) and cyst (**B**) at 40×; Trophozoites (**C**) and cyst (**D**) with higher magnification (60×). Images were obtained with ECHO Revolution.

In this study, the tephra sample was isolated and classified at the genotype level after analysis of the DF3 region of the 18S rDNA gene of *Acanthamoeba.* The obtained sequence has been deposited in the GenBank database under the following accession number: PP957191. The current sequence presented a ≥96% of homology with previously reported strains of *Acanthamoeba* genotype T4 recorded in the GenBank database. The phylogenetic analysis was based on the 18S partial sequence from different *Acanthamoeba* strains, which were aligned by ClustalW 2.0 software. The evolutionary history was inferred by using the MEGA 11 program and the maximum likelihood method, and *Balamuthia mandrillaris* isolate V039 was used as outgroup. From this phylogenetic analysis, we have obtained a drawn-to-scale tree (after a 500-replicates bootstrap test), where the relationship of the found strain with respect to the type sequences present in the GenBank database (Figure 4) is observed. In both solid and liquid culture mediums, trophozoites and cysts consistent with the genus *Acanthamoeba* were observed. The trophozoites isolated in this work presented a typically morphology with numerous vacuoles, lobulated acanthopodia, and a size of 15–30 μm [13,33] (Figure 5A,C). 

According to the historical subgenus classification groups of *Acanthamoeba* by Pussard and Pons [34], the cysts observed in this study were included in the Group II. This group is characterized by medium cysts with wrinkled, irregular ectocysts and polygonal stellate endocysts. The cyst showed a size ranged from 10 to 15 μm (Figure 5B,D).

### 3.3. Tolerance Characterization

Thermotolerance and osmotolerance studies were used to determine the strain’s potential pathogenicity. The obtained results revealed that the isolated strain showed excellent growth at room temperature (26 °C) and at 37 °C, but it was able to grow at a low concentration (for example, 0.1 M) and even at a medium concentration (1 M of mannitol). At 42 °C, we were only able to observe the cyst stage. However, at 1.5 M of mannitol, the cells were not capable of producing cysts (Table 2).

## 4. Discussion

*Acanthamoeba* is an opportunistic protozoan that has been described in various environments in nature, for example, in the nasal mucosa of healthy individuals [35], in water, soil, dust, and even the air [36]. Globally, *Acanthamoeba*-related illnesses such GAE, AK, and cutaneous infections are primarily caused by the T4 genotype. Due to its widespread dispersion in environmental sources and the resilience of cysts to disinfectants, this genotype has grown in scientific significance [37]. The genotype T4 has been found in soil from high-altitude mountains [38], but it has never been reported in volcanic tephra soil before. The presence of *Acanthamoeba* spp. in the volcanic archipelago of the Canary Islands is widely reported not only in water sources but also in a large variety of soils [24,25,39,40]. However, the previously analyzed soils have never belonged to a recent volcanic eruption such as the one evaluated in the present study. The identification of the T4 genotype was carried out using molecular techniques based on the analysis of DNA sequences, especially the 18S rRNA subunit gene (rns). It is a more specific and accurate tool compared to morphological classification since the latter can be subjective and depends on the interpretation of the observer, whereas genotyping is based on objective DNA sequence data. 

It is known that tephra deposits affect different aspects of vegetation recovery [41,42], completely resetting the seed bank depending on the depth of the tephra deposits [43]. Furthermore, the tephra layer will likely affect the water cycle, nutrient availability in the affected environments, and leaching volcanic compounds in the long term [44,45]. Consequently, the harsh conditions and the lack of nutrients of the evaluated tephra sample have been evidenced. Tephra is formed via the fragmentation of magma in the volcanic vent by bursting bubbles. The bubbles are related to volatile exsolution as magma rises with lower pressures in the vent. There are two main types of tephra deposits: those that form visible layers and tephra (mainly shards); and those that are not visible, which are known as cryptotephra [46]. Delaine and colleagues reported in 2016 [46] how the testate amoebae can collect and sort tephra particles, which become part of their shells in environments affected by the ash deposit after volcanic eruptions. 

The chemical and physical composition of the tephra material form this study, coupled with its recent volcanic origin, poses significant challenges for organism colonization. The combination of a coarse texture with low water retention and limited nutrient availability added to the presence of heavy metals creates an inhospitable environment for most life forms. Even though it is a harsh environment, it can be colonized by bacteria (mainly lithotrophic bacteria). Indeed, studies have shown early colonization of volcanic soils by various bacteria in the first few months after an eruption [47,48]. In summary, volcanic materials can harbor a great diversity of microorganisms within a few years of deposition [49]. This context may explain the presence of free-living amoebae in tephra since they can feed on the possible bacteria present in this environment. For that reason, the potential isolation of free-living amoebae in the material is particularly noteworthy, indicating the remarkable adaptability and survival capability of these organisms under extreme conditions. Moreover, this interaction between FLA and bacteria may indicate an increase in virulence in infections [50]. This highlights the existence of horizontal gene transfer (HGT). HGT has played an important role in the evolution of pathogenicity in *Acanthamoeba* [51]. To the best of our knowledge, this is the first survey of soil from volcanic material on the island La Palma and the first report on identifying genotype T4 from a tephra sample in the study area.

Several studies have reported the genotype T4 as the dominant genotype in soil sources. Moreover, this genotype is one of the main causes of amoebic keratitis, brain encephalitis, and skin infections [52,53]. Consequently, this FLA has been considered one of the most adaptable of the FLA group because it is able to survive in extreme environments. Celis and colleagues have isolated *Acanthamoeba* strains from an 80 °C volcanic mud spring water source in the Philippines. Considering that *Acanthamoeba* spp. was not known to be able to persist at temperatures greater than 65 °C damp heat until recently [54], these results contribute further knowledge on these opportunistic pathogens. 

In addition, only thermophilic FLA may be pathogenic for humans and animals, which is correlated with their adaptation to become capable of surviving in 37 °C. Therefore, the isolated strain is thermophilic because of its abundant growth at 26 °C and 37 °C, although at 42 °C, only viable cysts remain. However, it is not only pathogenic strains that are thermophilic [55,56], and for this reason, the thermal tolerance test is not sufficient to determine the pathogenicity of the detected strain. More accurate information regarding the organism’s capacity to infect humans and animals is needed. Several other methods of finding out the pathogenicity of amoebae have been described, such as osmotolerance assays. Osmotolerance indicates the ability of strains to adapt to tissues [57], and some studies relate these physiological constraints to the virulence of environmental strains [58]. In fact, we have demonstrated the organism’s moderate ability to grow at concentrations of up to 1 M mannitol and its inability to grow at 1.5 M concentrations. The pathogenic capacity of *Acanthamoeba* is multifactorial, and its ability to adapt to the protective osmolarity of the tears of the eye favors cytotoxicity, although there are other factors, such as the ability of the amoebae to produce proteases, which favor adhesion to the cells of the corneal epithelium [59].

Overall, in the current study, early findings on the existence of a potentially pathogenic *Acanthamoeba* strain, isolated in a harsh environment (in this case, newly volcanic soil), are presented.

## Figures and Tables

**Table 1:** Physicochemical characteristics of the tephra sample.

Parameter					
Gravel%	36.2	Fe_2_O_3_%	6.18	Cd ppm	356
Sand%	63.5	MnO%	0.12	Co ppm	209
Very coarse silt%	0.3	MgO%	2.85	Ni ppm	479
EC_1:5_ µS cm^−1^	114.7	CaO%	5.55	Cu ppm	213
pH_1:5_	6.9	Na_2_O%	3.22	Zn ppm	78
OC%	0.032	K_2_O%	1.43	Cr ppm	659
N%	0.005	P_2_O_5_%	0.00	Mo ppm	85
SiO_2_%	26.31	S ppm	1267	B ppm	0
Al_2_O_3_%	9.45	Pb ppm	1917		

**Table 2:** Behaviour of *Acanthamoeba* sp. T4 strain trophozoites and cysts incubated at different temperatures and exposed to different concentrations of mannitol (+++: abundant growth on the entire plate of Trophozoites (T)/cysts I; ++: moderate growth of T/C; +: mild growth of T/C; −: negative growth).

Cell Stage	Temperature Growth			Mannitol Growth		
	26 °C	37 °C	42 °C	0.1 M	1 M	1.5 M
Trophozoites	+++	++	−	+++	++	−
Cysts	+	++	++	+	++	−

## Data Availability

Sequence data are deposited in Genebank database. Any other data are available upon request to authors.
